# Risk factors for nosocomial infection in patients undergoing extracorporeal membrane oxygenation support treatment: A systematic review and meta-analysis

**DOI:** 10.1371/journal.pone.0308078

**Published:** 2024-11-25

**Authors:** Xiangui Lv, Yan Han, Daiqiang Liu, Xinwei Chen, Lvlin Chen, Huang Huang, Chao Huang

**Affiliations:** 1 Department of Intensive care medicine, Affiliated Hospital of Chengdu University, Chengdu, Sichuan, China; 2 Geriatrics Center of Affiliated Hospital of Chengdu University, Chengdu, Sichuan, China; 3 Department of Infection, Affiliated Hospital of Chengdu University, Chengdu, Sichuan, China; University of Pittsburgh Medical Center Pinnacle Health Medical Services, UNITED STATES OF AMERICA

## Abstract

**Objective:**

To evaluate the risk factors of nosocomial infection during Extracorporeal membrane oxygenation (ECMO) treatment through systematic evaluation and meta-analysis, in order to provide evidence-based basis for clinical treatment and prevention of nosocomial infection during ECMO treatment.

**Method:**

Computer search of Cochrane Library, PubMed, Embase, and Web of Science databases was conducted to establish a database of relevant literature published in March 2023. Two researchers independently screened literature, extracted data, and evaluated quality based on inclusion and exclusion criteria, and then analyzed the data using STATA 14.0 software. This plan is registered with PROSPERO as CRD42021271083.

**Result:**

A total of 2955 ECMO patients, including 933 nosocomial infected patients, were included in 23 articles. Meta analysis showed that immunosuppression, Heart transplantation, VA-ECMO, CRRT, red blood cell input, ECMO support time, mechanical ventilation time, ICU hospitalization time, and total hospitalization time were the risk factors for nosocomial infection in patients supported by ECMO.

**Conclusion:**

ECMO treatment for nosocomial infections in patients is related to multiple factors. In clinical work, medical staff should identify high-risk groups of ECMO nosocomial infections, actively take preventive measures, and reduce the incidence and mortality of nosocomial infections.

## Introduction

The World Health Organization currently defines nosocomial infection as infections that occur during the care process of a patient in a hospital or other healthcare facility, are not present at admission, or are latent. Ventilator associated pneumonia (VAP) and Bloodstream infection (BSI) are common types of nosocomial infections in critically ill patients [1]. VAP is defined as pneumonia that occurs 48 hours after mechanical ventilation and is a common complication after tracheal intubation in critically ill patients [2]. BSI is defined by positive blood cultures in a patient with systemic signs of infection and may be either secondary to a documented source or primary—that is, without identified origin [3]. Nosocomial infection is one of the most common complications after Extracorporeal membrane oxygenation (ECMO) treatment, and is associated with increased demand for antibiotic treatment and higher nursing costs [1, 4]. Nosocomial infection is a global public health issue, with over 140000 patients worldwide dying from NI every year [5]. ECMO is an advanced respiratory and circulatory support technology widely used as an extracorporeal life support (ECLS) strategy for patients with respiratory and/or heart failure [6]. However, ECMO can cause various complications such as bleeding, thrombosis, and infection during use, leading to a significant increase in mortality rate [7]. A meta-analysis showed that the prevalence of nosocomial acquired infections was 8.8–64.0%, with a 4% increase in mortality risk. Nosocomial acquired infections have been identified as the most common complication among patients receiving ECMO, seriously affecting patient recovery [8]. Nosocomial infection has become a challenge for ECMO weaning. In order to effectively prevent the occurrence of nosocomial infection in patients receiving ECMO support treatment, many scholars have explored the risk factors of nosocomial infection in ECMO support treatment patients, but the results of various studies are inconsistent. At present, there is still a lack of meta-analysis on the risk factors of nosocomial infection in patients receiving ECMO support treatment. This article conducts a systematic evaluation and meta-analysis by collecting research on the risk factors of nosocomial infection in patients receiving ECMO support treatment, aiming to provide evidence-based basis for clinical prevention of nosocomial infection.

## Information and methods

Inclusion and Exclusion Criteria for Literature Inclusion Criteria:

Inclusion criteria: publicly published research on risk factors for nosocomial infection during ECMO treatment; The study type is Cohort study or Case–control study; The diagnostic criteria for nosocomial infection are clear; Study subjects were adult patients.

Exclusion criteria: Repeated publications; Incorrect data collection or statistical methods; Review, case reports, and animal experiments.

### Research retrieval strategy

A study on the risk factors of nosocomial infection during ECMO treatment, published on March 1, 2023, was conducted through computer retrieval of Cochrane Library, PubMed, Embase, Web of Science, and database creation to retrieval. Use the following keywords: “Extracorporeal Membrane Oxygenations/Membrane Oxygenation, Extracorporeal/ECMO Treatment/Extracorporeal Life Support” “Cross Infections/Hospital Infection/Nosocomial Infection”.

### Data extraction

Two researchers (Xiangui Lv and Xinwei Chen) independently completed literature screening and data extraction. If there are different opinions on the literature and data, they will negotiate with the third researcher (Chao Huang) to resolve the issue. The data extraction content includes the author of the included study, country, publication time, follow-up time, sample size, study type, nosocomial infection type, and influencing factors involved.

### Quality evaluation of studies

The Newcastle Ottawa Scale (NOS) evaluation criteria were used to evaluate the quality of literature. NOS includes three parts: study population selection, inter group comparability, and exposure factors, with a maximum score of 9 A score of 7 indicates high-quality research, a score of 5–6 indicates medium quality research, and a score of ≤ 4 indicates low-quality research. If there are differences of opinion during the evaluation process, they can be resolved through mutual discussion or consultation with the third researcher.

### Statistical analysis

Using STATA 14.0 software for meta-analysis of literature, heterogeneity testing was first conducted. If P>0.1 and I2<50%, it indicates good homogeneity between studies, and a fixed effects model was used for analysis; If P ≤ 0.1 and I2>50%, it indicates heterogeneity between studies, and a random effects model is used for analysis. Count data using OR as an outcome measure, calculate the combined OR value and its 95% CI. The continuous variable uses standardized mean deviation as the effect indicator.

## Results

### Study retrieval results

**Fig 1:**
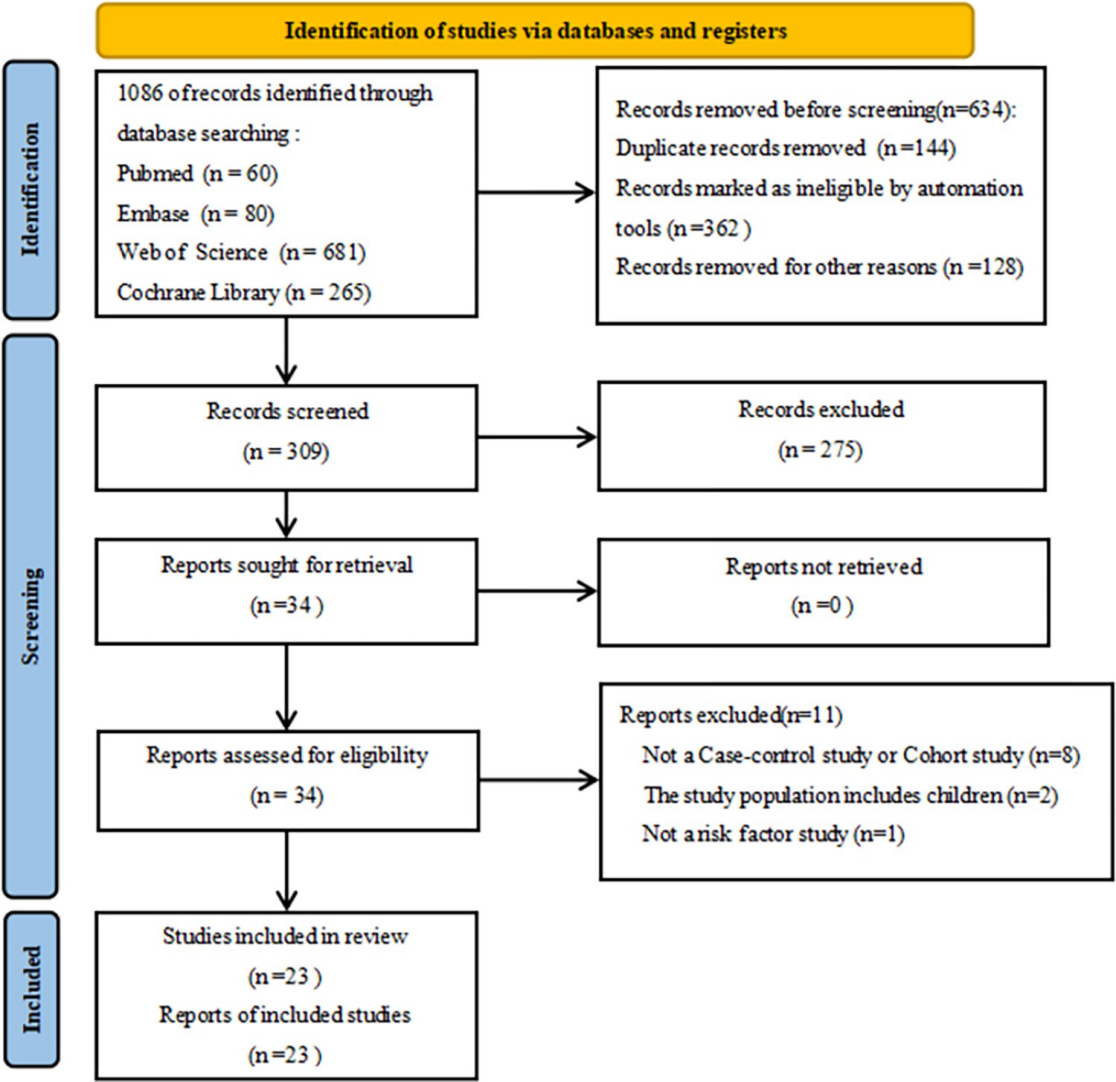
’Flowchart of’ study screening.

In the preliminary screening, a total of 1086 related studies were retrieved. According to the inclusion and exclusion criteria, a total of 23 studies were included, including 2955 ECMO patients, including 933 nosocomial infected patients. The flow chart of literature screening is shown in Fig 1. Basic characteristics of the included literature Table 1, and the results of literature quality evaluation are shown in Table 2

**Table 1:** Basic characteristics of included literature.

author	country	year	follow-up time	Patient type	Infection type	sample size	research design	Risk factors
Aubron [9]	Australia	2013	2005.01–2011.06	-	NI	146	Retrospective	(6) (7) (8) (9) (11) (12)
Austin [10]	Australia	2017	2011.04–2014.03	-	NI	99	Retrospective	(2) (4) (5) (6) (6) (7) (8) (9) (10) (13) (14)
Allou [11]	France	2019	2010.01–2016.12	-	CRI	220	Retrospective	(1) (2) (4) (6) (8) (9) (11) (12)
Burket [12]	America	1999	1985.02–1995.10	-	NI	71	Retrospective	(9) (10) (13) (14)
Bougle [13]	France	2018	2013.01–2014.12	-	VAP	152	Retrospective	(4) (8)
Hsu [14]	China	2009	2001.07–2007.06	-	NI	114	Retrospective	(6) (7)(8)(9) (12)(13) (14)
Juthani [15]	America	2018	2012.01–2015.07	-	NI	100	Retrospective	(6)(7)
Kim [16]	Korea	2016	2012.01–2014.08	-	NI	47	Retrospective	(6)(7)(9)(12) (13) (14)
Kim [17]	Korea	2017	2011.01–2015.12	-	NI	61	Retrospective	(9)
Ko [18]	Korea	2020	2010.01–2018.12	cardiac arrest	NI	150	Retrospective	(1)(2)(3) (5)(9)(13)
Kutlesa [19]	Croatia	2017	2009.10–2016.06	ARDS	NI	100	Prospective	(1) (4)(8)(9)
Li [20]	China	2021	2013.01–2019.12	-	NI	56	Retrospective	(6)(7)(9)(10) (14)
Li [21]	China	2018	2012.12–2015.08	Post cardiac surgery	NI	74	Retrospective	(6)(7)(9)(10) (13) (14)
Menaker [22]	America	2019	2010.03–2015.03	-	BSI	145	Retrospective	(9) (11)
Schmidt [23]	France	2012	2003.01–2009.12	cardiogenic shock	NI	220	Retrospective	(1)(4)(5)(12)
Sun [24]	China	2010	1996.01–2007.12	-	NI	334	Retrospective	(5)(6)(7)(9) (12)
Sun [25]	China	2017	2009.01–2014.03	-	NI	75	Retrospective	(4)(6)(7) (14)
Wang [26]	China	2020	2013.01–2019.08	-	HAP	69	Retrospective	(1)(7)(8)(9) (10)(11) (14)
Wang [27]	China	2021	2012.01–2017.12	post cardiac surgery	NI	322	Retrospective	(1)(2)(3)(8)(9)
Wang [28]	China	2020	2013.01–2019.08	-	BSI	69	Retrospective	(8)(9)(10) (14)
Wang[29]	China	2023	2015.01–2021.10	-	NI	196	Retrospective	(1)(2)(6)(7)(8) (9)(10) (13) (14)
Zhan [30]	China	2021	2013.01–2019.12	-	NI	56	Retrospective	(6)(7)(9)(10) (14)
Xu [31]	China	2022	2011.01–2020.09	Nonsurgical Patients	NI	79	Retrospective	(1) (6)(7)(9)(10) (13) (14)

NI: nosocomial infection; CRI: Catheter-related Infection; BSI:bloodstream infection; HAP: Hospital acquired pneumonia; VAP: ventilator associated pneumonia.

Risk factors: (1)Body Mass Index (BMI); (2) Intubation in the operating room; (3) Intubation in the ward; (4) Immunosuppression; (5) Heart transplant; (6)VA-ECMO; (7)VV-ECMO; (8) Continuous Renal Replacement Therapy(CRRT); (9) ECMO duration; (10) Duration of mechanical ventilation; (11) Number of red blood cell units infused; (12) Pre ECMO hospitalization time; (13) ICU hospitalization time; (14) Total hospitalization time.

**Table 2:** Bias risk assessment results.

	Study population selection				Comparability		Outcome			Total score
Author	Exposure group selection	Non exposure group selection	Method for determining exposure	There are no outcome events at the beginning of the study	Controlling major confounding factors	Controlling other confounding factors	Adequate evaluation	Adequate follow-up	Follow up integrity	
Aubron [9]	1	1	1	0	0	1	1	0	0	5
Austin [10]	1	1	1	0	1	0	1	0	0	5
Allou [11]	1	1	1	1	0	1	1	1	0	7
Burket [12]	1	1	1	1	0	0	1	0	0	5
Bougle [13]	1	1	1	1	1	1	1	0	0	7
Hsu [14]	1	1	1	1	0	1	1	0	0	6
Juthani [15]	1	1	1	0	0	1	1	0	0	5
Kim [16]	1	1	1	0	0	1	1	0	0	5
Kim [17]	1	1	1	0	0	1	1	1	0	6
Ko [18]	1	1	1	1	1	1	1	0	0	7
Kutlesa [19]	1	1	1	0	0	1	1	0	0	6
Li [20]	1	1	0	0	0	1	1	0	0	4
Li [21]	1	1	1	0	0	1	1	0	0	5
Menaker [22]	1	1	1	0	0	0	1	0	0	4
Schmidt [23]	1	1	1	1	0	1	1	1	0	7
Sun [24]	1	1	1	0	0	1	1	0	0	6
Sun [25]	1	1	1	0	0	1	1	0	0	6
Wang [26]	1	1	1	1	0	1	1	1	0	7
Wang [27]	1	1	1	1	0	1	1	0	0	6
Wang [28]	1	1	1	0	0	1	1	0	0	5
Wang [29]	1	1	1	0	0	1	1	0	0	5
Zhan [30]	1	1	1	1	0	1	1	1	0	7
Xu [31]	1	1	1	0	0	1	1	0	0	5

### Basic characteristics and bias risk assessment results included in the study

Of the 23 included studies, 22 were retrospective studies and 1 was prospective Cohort study; Published from 1999 to 2023; Distributed in China, the United States, South Korea, France, Australia and Croatia; Five studies with a NOS score of ≥ 7, 16 studies with a score of 5–6, and 2 studies with a score of ≤ 4 were included in the study. Most studies were of medium quality. The basic characteristics of the included study are shown in Table 1, and the results of the risk assessment of bias in the included study are shown in Table 2.

### Meta analysis of risk factors for nosocomial infection during ECMO treatment

A total of 34 risk factors were mentioned in 23 articles, of which ≥ 2 articles involved a total of 14 factors. A meta-analysis of 14 risk factors showed that immunosuppression, heart transplantation, VA-ECMO, ECMO support time, mechanical ventilation time, CRRT, red blood cell input, ICU hospitalization time, and total hospitalization time were risk factors for nosocomial infections. The results of the meta-analysis are shown in Table 3.

**Table 3:** Meta analysis of risk factors for nosocomial infection during ECMO treatment.

Risk factors	Number of studies	Infection		heterogeneity test		analysis model	OR/SMD	95%CI	P	Egger’s test
		YES	NO	I^2^	P					
BMI	7	482	795	0	0.845	fixed	0.12	-0.00∽0.25	0.058	0.092
immunosuppression	8	420	671	0	0.935	fixed	1.60	1.18∽2.17	0.003	0.324
Heart transplant	4	243	560	0	0.432	fixed	3.37	1.95∽5.82	<0.001	0.650
VV-ECMO	13	375	1070	58.9	0.004	Random	0.79	0.50∽1.25	0.319	0.506
VA-ECMO	12	358	1159	82.0	<0.001	random	1.79	1.10∽2.93	0.020	0.641
Intubation in the operating room	4	226	565	0	0.876	fixed	0.96	0.71∽1.28	0.771	0.294
Intubation in the ward	3	166	306	0.0	0.998	fixed	1.07	0.78∽1.47	0.672	0.266
ECMO duration	20	680	1803	71.5	<0.001	random	0.78	0.59∽0.97	<0.001	0.120
Duration of mechanical ventilation	9	283	486	78.4	<0.001	random	0.66	0.31∽1.01	<0.001	0.115
CRRT	11	1039	460	0.0	0.515	fixed	1.39	1.14∽1.69	0.001	0.599
Number of red blood cell units infused	4	108	472	0	0.682	fixed	0.42	0.21∽0.62	<0.001	0.767
Pre ECMO hospitalization time	6	809	286	47	0.093	random	0.12	-0.10∽0.34	0.300	0.885
ICU hospitalization time	6	517	234	77.8	<0.001	random	0.55	0.18∽0.92	0.004	0.399
Total hospitalization time	10	679	326	78.2	<0.001	random	0.75	0.43∽1.07	<0.001	0.582

### Other risk factors

In this study, multiple risk factors were only reported in a single literature and cannot be combined with meta-analysis for descriptive analysis. Elevated creatinine levels [17]、Cardiopulmonary resuscitation pumped on time [18]、Bleeding (over 1000ml per transfusion) [19]、ECMO duration>250h[19]、liver failure [22]、renal failure [22]、mechanical complications include oxygenator failure [24]、autoimmune diseases (including systemic lupus erythematosus, Wegener’s granulomatosis, Dermatomyositis, antiphospholipid syndrome and Scleroderma with Vasculitis, etc.) [24]、viral pneumonia [31]、high Sequential Organ Failure Assessment (SOFA) score [28] are risk factors for nosocomial infection in ECMO patients.

### Publication bias test

Egger’s test has been used for publication bias testing, and the results indicate that there is no publication bias. The Eggers test results are shown in Table 3

## Discussion

Based on the published Cohort study and Case–control study, this Systematic review and meta-analysis explored the potential risk factors of nosocomial infection in patients supported by ECMO, and included subjects from 23 studies. We found that the potential risk factors for nosocomial infection in patients with ECMO support were immunosuppression, Heart transplantation, VA-ECMO, ECMO support time, mechanical ventilation time, CRRT, red blood cell input, ICU hospital stay, and total hospital stay.

There is a correlation between ECMO support time and nosocomial infection. ECMO support for more than 250 hours can significantly increase the incidence rate of BSI. Therefore, it is necessary to carefully evaluate the symptoms and signs of invasive instruments, intubation site wounds, and fever. If the patient has persistent fever and increased white blood cells, especially when the duration of ECMO exceeds 10 days, a complete assessment of sepsis should be conducted [14, 19]. Prolonged mechanical ventilation time is a risk factor for nosocomial infection. During mechanical ventilation treatment, tracheal intubation can damage the natural protective barrier of the respiratory tract, weaken tracheal ciliary movement and cough reflex, which increases the risk of pathogen invasion and further increases the risk of VAP occurrence. [32]. In patients with long-term mechanical ventilation, the respiratory function is significantly degraded, the Pharyngeal reflex and the ability to cough and expectorate are weakened, and the pathogenic bacteria colonized in the oropharynx can invade the lungs through mechanical ventilation, causing infection [33]. Tracheostomy in patients with tracheal intubation can reduce airflow resistance, respiratory force, and sedation needs, while improving patient comfort and mobility [34]. Early tracheostomy in ECMO patients is significantly associated with good patient prognosis, as well as shortened mechanical ventilation, ECMO operation, and ICU hospitalization time [35, 36]. The implementation of tracheostomy during ECMO operation can reduce the incidence of hospital infections and improve patient outcomes.

The prolonged hospitalization time and total hospitalization time in ICU are risk factors for nosocomial infection, which may be related to the more critical condition of patients with prolonged hospitalization time, the relative increase in various treatments and invasive procedures, and the increased chance of infection. When the patient’s condition allows, the length of hospitalization should be minimized as much as possible.

CRRT treatment is a risk factor for nosocomial infections in patients receiving ECMO. ECMO and CRRT support treatment increased significant variability in antibiotic blood concentration and hemodynamic changes [13]. Bizarro and AUSTIN’s research have similar results, with VA ECMO having a higher incidence of nosocomial infections than VV ECMO [10, 37]. Possible reason is related to wound infection at the site of arterial catheterization in VA ECMO patients [10]. However, some studies have found that VV ECMO mode has a higher likelihood of VAP occurrence, which may be due to patients receiving VV ECMO receiving longer ECMO treatment and mechanical ventilation time [26]. Heart transplantation and immunosuppression are risk factors for nosocomial infection in ECMO patients. Heart transplantation is an effective method to treat end-stage heart disease. However, due to preoperative heart failure, it is often accompanied by multiple organ dysfunction. After a long period of general anesthesia, extracorporeal circulation and other invasive operations, steroids and other Immunosuppressive drug after transplantation may affect wound healing and increase the risk of infection. Therefore, medical staff should do a good job in monitoring the nosocomial infection of ECMO patients after Heart transplantation, which is conducive to the timely detection, diagnosis, treatment and supervision of nosocomial infection.

Blood transfusion products contain various immune neurotransmitters, such as residual white blood cells, aged red blood cells, residual platelets, etc. These immune neurotransmitters can promote the release of anti-inflammatory cytokines, leading to a decrease in neutrophil and natural killer cell activity, inducing immune suppression and increasing the risk of nosocomial infections in patients, which has a negative impact on the prognosis of critically ill patients [38–40]. In recent years, some studies have recommended restrictive transfusion strategies, which patients can benefit from. Implementing restrictive blood transfusion strategies may potentially reduce the incidence of nosocomial infections. Studies have shown that starting blood transfusions in ECMO patients with Hb<7 g/dL did not lead to an increase in patient mortality [40–42].

This review provides a reference for the prevention of nosocomial infections in patients treated with ECMO. In clinical work, medical staff should identify high-risk groups of ECMO nosocomial infections, actively take preventive measures, and reduce the incidence and mortality of nosocomial infections. At the same time, in the future research, more high-quality multi center prospective Cohort study is needed to evaluate the risk factors of nosocomial infection in ECMO patients more scientifically and comprehensively, so as to provide reliable basis for the prevention of nosocomial infection in ECMO patients. It is undeniable that this study has some limitations. Firstly, the baseline differences among the included subjects are significant, and the risk factors interact with each other. However, due to the limited number of included studies, detailed subgroup analysis cannot be conducted, which may affect the effectiveness of statistical analysis. Secondly, it is not possible to calculate the estimated effects of all risk factors, as only two or more studies related to nosocomial infection risk factors with the same definition were summarized in the meta-analysis. Finally, the quality of the included literature varies, with some studies having small sample sizes, which affects the accuracy of the results and may limit the universality of the research results. However, our research results have made an important contribution to determining the risk factors of nosocomial infection in ECMO patients by integrating studies involving ECMO nosocomial infection risk factors.

## Conclusion

In summary, this study shows that immunosuppression, heart transplantation, VA-ECMO, ECMO support time, mechanical ventilation time, CRRT, red blood cell input, ICU hospitalization time, and total hospitalization time are risk factors for nosocomial infections in ECMO treated patients. In clinical work, medical staff should identify high-risk populations for ECMO nosocomial infection, actively take preventive measures, and reduce the incidence and mortality rate of nosocomial infection. Due to the limitations of inclusion in the research institute, the above conclusions still need to be further validated through large-scale, high-quality prospective cohort studies.

## Supporting information

S1 Appendix PRISMA checklist. (PDF)

S2 Appendix Search strategy. (DOCM)

S1 Data (XLSX)

## References

[1] Walter J, Haller S, Quinten C, Kärki T, Zacher B, Eckmanns T, et al. Healthcare-associated pneumonia in acute care hospitals in European Union/European Economic Area countries: an analysis of data from a point prevalence survey, 2011 to 2012. Euro surveillance: bulletin Europeen sur les maladies transmissibles = European communicable disease bulletin. 2018;23(32). Epub 2018/08/16. doi: 10.2807/1560-7917.ES.2018.23.32.1700843 ; PubMed Central PMCID: PMC6092912. 30107871 PMC6092912

[2] Kalanuria AA, Ziai W, Mirski M. Ventilator-associated pneumonia in the ICU. Critical care (London, England). 2014;18(2):208. Epub 2014/07/17. doi: 10.1186/cc13775 ; PubMed Central PMCID: PMC4056625. 25029020 PMC4056625

[3] Timsit JF, Ruppé E, Barbier F, Tabah A, Bassetti M. Bloodstream infections in critically ill patients: an expert statement. Intensive care medicine. 2020;46(2):266–84. Epub 2020/02/13. doi: 10.1007/s00134-020-05950-6 ; PubMed Central PMCID: PMC7223992. 32047941 PMC7223992

[4] Rodríguez-Acelas AL, de Abreu Almeida M, Engelman B, Cañon-Montañez W. Risk factors for health care-associated infection in hospitalized adults: Systematic review and meta-analysis. American journal of infection control. 2017;45(12):e149–e56. Epub 2017/10/17. doi: 10.1016/j.ajic.2017.08.016 . 29031433

[5] Blot S, Ruppé E, Harbarth S, Asehnoune K, Poulakou G, Luyt CE, et al. Healthcare-associated infections in adult intensive care unit patients: Changes in epidemiology, diagnosis, prevention and contributions of new technologies. Intensive & critical care nursing. 2022;70:103227. Epub 2022/03/08. doi: 10.1016/j.iccn.2022.103227 ; PubMed Central PMCID: PMC8892223. 35249794 PMC8892223

[6] Wrisinger WC, Thompson SL. Basics of Extracorporeal Membrane Oxygenation. Surg Clin North Am. 2022;102(1):23–35. Epub 2021/11/21. doi: 10.1016/j.suc.2021.09.001 ; PubMed Central PMCID: PMC8598290. 34800387 PMC8598290

[7] Corley A, Lye I, Lavana JD, Ahuja A, Anstey CM, Jarrett P, et al. Nosocomial infection prevalence in patients undergoing extracorporeal membrane oxygenation (ECMO): protocol for a point prevalence study across Australia and New Zealand. BMJ Open. 2019;9(7):e029293. Epub 2019/07/13. doi: 10.1136/bmjopen-2019-029293 ; PubMed Central PMCID: PMC6624104. 31296512 PMC6624104

[8] Li X, Wang L, Wang H, Hou X. Outcome and Clinical Characteristics of Nosocomial Infection in Adult Patients Undergoing Extracorporeal Membrane Oxygenation: A Systematic Review and Meta-Analysis. Front Public Health. 2022;10:857873. Epub 2022/07/12. doi: 10.3389/fpubh.2022.857873 ; PubMed Central PMCID: PMC9268548. 35812481 PMC9268548

[9] Aubron C, Cheng AC, Pilcher D, Leong T, Magrin G, Jamie Cooper D, et al. Infections acquired by adults who receive extracorporeal membrane oxygenation: Risk factors and outcome. Infect Control Hosp Epidemiol. 2013;34(1):24–30. doi: 10.1086/668439 23221189

[10] Austin DE, Kerr SJ, Al-Soufi S, Connellan M, Spratt P, Goeman E, et al. Nosocomial infections acquired by patients treated with extracorporeal membrane oxygenation. Crit Care Resusc. 2017;19(Suppl 1):68–75. Epub 2017/11/01. . 29084504

[11] Allou N, Lo Pinto H, Persichini R, Bouchet B, Braunberger E, Lugagne N, et al. Cannula-Related Infection in Patients Supported by Peripheral ECMO: Clinical and Microbiological Characteristics. Asaio j. 2019;65(2):180–6. Epub 2018/03/09. doi: 10.1097/MAT.0000000000000771 . 29517513

[12] Burket JS, Bartlett RH, Vander Hyde K, Chenoweth CE. Nosocomial infections in adult patients undergoing extracorporeal membrane oxygenation. Clin Infect Dis. 1999;28(4):828–33. Epub 2000/05/29. doi: 10.1086/515200 . 10825046

[13] Bouglé A, Bombled C, Margetis D, Lebreton G, Vidal C, Coroir M, et al. Ventilator-associated pneumonia in patients assisted by veno-arterial extracorporeal membrane oxygenation support: Epidemiology and risk factors of treatment failure. PLoS One. 2018;13(4):e0194976. Epub 2018/04/14. doi: 10.1371/journal.pone.0194976 ; PubMed Central PMCID: PMC5898723. 29652913 PMC5898723

[14] Hsu MS, Chiu KM, Huang YT, Kao KL, Chu SH, Liao CH. Risk factors for nosocomial infection during extracorporeal membrane oxygenation. J Hosp Infect. 2009;73(3):210–6. Epub 2009/09/29. doi: 10.1016/j.jhin.2009.07.016 . 19782430

[15] Juthani BK, Macfarlan J, Wu J, Misselbeck TS. Incidence of nosocomial infections in adult patients undergoing extracorporeal membrane oxygenation. Heart Lung. 2018;47(6):626–30. Epub 2018/09/01. doi: 10.1016/j.hrtlng.2018.07.004 . 30166066

[16] Kim DW, Yeo HJ, Yoon SH, Lee SE, Lee SJ, Cho WH, et al. Impact of bloodstream infections on catheter colonization during extracorporeal membrane oxygenation. J Artif Organs. 2016;19(2):128–33. Epub 2016/01/02. doi: 10.1007/s10047-015-0882-5 . 26721824

[17] Kim GS, Lee KS, Park CK, Kang SK, Kim DW, Oh SG, et al. Nosocomial Infection in Adult Patients Undergoing Veno-Arterial Extracorporeal Membrane Oxygenation. J Korean Med Sci. 2017;32(4):593–8. Epub 2017/03/01. doi: 10.3346/jkms.2017.32.4.593 ; PubMed Central PMCID: PMC5334156. 28244284 PMC5334156

[18] Ko RE, Huh K, Kim DH, Na SJ, Chung CR, Cho YH, et al. Nosocomial infections in in-hospital cardiac arrest patients who undergo extracorporeal cardiopulmonary resuscitation. PLoS One. 2020;15(12):e0243838. Epub 2020/12/29. doi: 10.1371/journal.pone.0243838 ; PubMed Central PMCID: PMC7757900. 33362276 PMC7757900

[19] Kutleša M, Santini M, Krajinović V, Papić N, Novokmet A, Josipović Mraović R, et al. Nosocomial blood stream infections in patients treated with venovenous extracorporeal membrane oxygenation for acute respiratory distress syndrome. Minerva Anestesiol. 2017;83(5):493–501. Epub 2017/01/27. doi: 10.23736/S0375-9393.17.11659-7 . 28124861

[20] Li ZJ, Zhang DF, Zhang WH. Analysis of Nosocomial Infection and Risk Factors in Patients with ECMO Treatment. Infect Drug Resist. 2021;14:2403–10. Epub 2021/07/03. doi: 10.2147/IDR.S306209 ; PubMed Central PMCID: PMC8241808. 34211285 PMC8241808

[21] Li BF, Sun GQ, Cheng Z, Mei CC, Liao XZ, Li JW, et al. Analysis of Nosocomial Infections in Post-Cardiac Surgery Extracorporeal Membrane Oxygenation Support Therapy. Heart Surg Forum. 2018;21(5):E387–E91. doi: 10.1532/hsf.1789 WOS:000457932600011. 30311890

[22] Menaker J, Galvagno S, Rabinowitz R, Penchev V, Hollis A, Kon Z, et al. Epidemiology of blood stream infection in adult extracorporeal membrane oxygenation patients: A cohort study. Heart Lung. 2019;48(3):236–9. Epub 2019/01/29. doi: 10.1016/j.hrtlng.2019.01.004 . 30686618

[23] Schmidt M, Bréchot N, Hariri S, Guiguet M, Luyt CE, Makri R, et al. Nosocomial Infections in Adult Cardiogenic Shock Patients Supported by Venoarterial Extracorporeal Membrane Oxygenation. Clinical Infectious Diseases. 2012;55(12):1633. 1188335404. doi: 10.1093/cid/cis783 22990851 PMC3888098

[24] Sun HY, Ko WJ, Tsai PR, Sun CC, Chang YY, Lee CW, et al. Infections occurring during extracorporeal membrane oxygenation use in adult patients. J Thorac Cardiovasc Surg. 2010;140(5):1125–32.e2. Epub 2010/08/17. doi: 10.1016/j.jtcvs.2010.07.017 . 20708754

[25] Sun GQ, Li BF, Lan HL, Wang J, Lu LF, Feng XQ, et al. Risk factors for nosocomial infections in patients receiving extracorporeal membrane oxygenation supportive therapy. Medicina Clinica. 2017;149(10):423–8. doi: 10.1016/j.medcli.2017.03.038 WOS:000415030800001. 28647277

[26] Wang J, Huang J, Hu W, Cai X, Hu W, Zhu Y. Risk factors and prognosis of nosocomial pneumonia in patients underg-oing extracorporeal membrane oxygenation: a retrospective study. J Int Med Res. 2020;48(10):300060520964701. Epub 2020/10/23. doi: 10.1177/0300060520964701 ; PubMed Central PMCID: PMC7585896. 33086927 PMC7585896

[27] Wang J, Wang L, Jia M, Du Z, Hou X. Extracorporeal Membrane Oxygenation-Related Nosocomial Infection after Cardiac Surgery in Adult Patients. Braz J Cardiovasc Surg. 2021;36(6):743–51. Epub 2021/02/13. doi: 10.21470/1678-9741-2020-0068 ; PubMed Central PMCID: PMC8641764. 33577254 PMC8641764

[28] Wang JR, Huang JY, Hu W, Cai XY, Hu WH, Zhu Y. Bloodstream infections in patients undergoing extracorporeal membrane oxygenation. Pak J Med Sci. 2020;36(6):1171–6. Epub 2020/09/25. doi: 10.12669/pjms.36.6.2882 ; PubMed Central PMCID: PMC7501021. 32968375 PMC7501021

[29] Wang L, Ni K, Wang Y, Lu H, Fang J, Chen C. Nosocomial Infections in Adult Patients Receiving Extracorporeal Membrane Oxygenation in China: A Retrospective Cohort Study. Am J Infect Control. 2023. Epub 2023/04/15. doi: 10.1016/j.ajic.2023.04.010 . 37059121

[30] Zhan-Jie L, Dong-Fang Z, Wei-Hong Z. Analysis of Nosocomial Infection and Risk Factors in Patients with ECMO Treatment. Infect Drug Resistance. 2021;14:2403–10. doi: 10.2147/IDR.S306209 2547128472. 34211285 PMC8241808

[31] Xu W, Fu Y, Yao Y, Zhou J, Zhou H. Nosocomial Infections in Nonsurgical Patients Undergoing Extracorporeal Membrane Oxygenation: A Retrospective Analysis in a Chinese Hospital. Infect Drug Resist. 2022;15:4117–26. Epub 2022/08/09. doi: 10.2147/IDR.S372913 ; PubMed Central PMCID: PMC9347224. 35937786 PMC9347224

[32] Wałaszek M, Kosiarska A, Gniadek A, Kołpa M, Wolak Z, Dobroś W, et al. The risk factors for hospital-acquired pneumonia in the Intensive Care Unit. Przegl Epidemiol. 2016;70(1):15–20, 107–10. Epub 2016/06/28. . 27344468

[33] Papazian L, Klompas M, Luyt CE. Ventilator-associated pneumonia in adults: a narrative review. Intensive Care Med. 2020;46(5):888–906. Epub 2020/03/12. doi: 10.1007/s00134-020-05980-0 ; PubMed Central PMCID: PMC7095206. 32157357 PMC7095206

[34] Hosokawa K, Nishimura M, Egi M, Vincent JL. Timing of tracheotomy in ICU patients: a systematic review of randomized controlled trials. Crit Care. 2015;19:424. Epub 2015/12/05. doi: 10.1186/s13054-015-1138-8 ; PubMed Central PMCID: PMC4669624. 26635016 PMC4669624

[35] DiChiacchio L, Boulos FM, Brigante F, Raithel M, Shah A, Pasrija C, et al. Early tracheostomy after initiation of venovenous extracorporeal membrane oxygenation is associated with decreased duration of extracorporeal membrane oxygenation support. Perfusion. 2020;35(6):509–14. Epub 2020/02/06. doi: 10.1177/0267659119898327 . 32020840

[36] Nukiwa R, Uchiyama A, Tanaka A, Kitamura T, Sakaguchi R, Shimomura Y, et al. Timing of tracheostomy and patient outcomes in critically ill patients requiring extracorporeal membrane oxygenation: a single-center retrospective observational study. J Intensive Care. 2022;10(1):56. Epub 2022/12/31. doi: 10.1186/s40560-022-00649-w ; PubMed Central PMCID: PMC9802016. 36585705 PMC9802016

[37] Bizzarro MJ, Conrad SA, Kaufman DA, Rycus P. Infections acquired during extracorporeal membrane oxygenation in neonates, children, and adults. Pediatr Crit Care Med. 2011;12(3):277–81. Epub 2010/05/25. doi: 10.1097/PCC.0b013e3181e28894 . 20495508

[38] Muszynski JA, Spinella PC, Cholette JM, Acker JP, Hall MW, Juffermans NP, et al. Transfusion-related immunomodulation: review of the literature and implications for pediatric critical illness. Transfusion. 2017;57(1):195–206. Epub 2016/10/04. doi: 10.1111/trf.13855 ; PubMed Central PMCID: PMC8341144. 27696473 PMC8341144

[39] Ghio M, Contini P, Negrini S, Mazzei C, Zocchi MR, Poggi A. Down regulation of human natural killer cell-mediated cytolysis induced by blood transfusion: role of transforming growth factor-β(1), soluble Fas ligand, and soluble Class I human leukocyte antigen. Transfusion. 2011;51(7):1567–73. Epub 2011/01/11. doi: 10.1111/j.1537-2995.2010.03000.x . 21214580

[40] Salpeter SR, Buckley JS, Chatterjee S. Impact of more restrictive blood transfusion strategies on clinical outcomes: a meta-analysis and systematic review. Am J Med. 2014;127(2):124–31.e3. Epub 2013/12/18. doi: 10.1016/j.amjmed.2013.09.017 . 24331453

[41] Voelker MT, Busch T, Bercker S, Fichtner F, Kaisers UX, Laudi S. Restrictive transfusion practice during extracorporeal membrane oxygenation therapy for severe acute respiratory distress syndrome. Artif Organs. 2015;39(4):374–8. Epub 2014/10/29. doi: 10.1111/aor.12385 . 25349127

[42] Martucci G, Schmidt M, Agerstrand C, Tabatabai A, Tuzzolino F, Giani M, et al. Transfusion practice in patients receiving VV ECMO (PROTECMO): a prospective, multicentre, observational study. Lancet Respir Med. 2023;11(3):245–55. Epub 2022/10/15. doi: 10.1016/S2213-2600(22)00353-8 . 36240836

