# Supplementary material for: Risk factors for nosocomial infection in patients undergoing extracorporeal membrane oxygenation support treatment: A systematic review and meta-analysis
Source: PLoS One. 2024 Nov 25;19(11):e0308078. doi: 10.1371/journal.pone.0308078 (PMC11588223; doi:10.1371/journal.pone.0308078)
Supplement: S2 Appendix — (DOCM) [file pone.0308078.s002.docm]

**S2 Appendix. Search strategy**

**Pubmed**

| #1 | "extracorporeal life support"[Title/Abstract] OR  "ECMO"[Title/Abstract] OR "extracorporeal membrane oxygenation"[Title/Abstract] | 20621 |
| --- | --- | --- |
| #2 | "healthcare associated infection"[Title/Abstract] OR "nosocomial infection"[Title/Abstract] OR "hospital infections"[Title/Abstract] | 10455 |
|  | #1 AND #2 | 60 |

**Embase**

| #1 | 'extracorporeal membrane oxygenation':ab,ti OR 'ecmo':ab,ti OR 'extracorporeal life support':ab,ti | 34954 |
| --- | --- | --- |
| #2 | 'healthcare associated infection':ab,ti OR 'nosocomial infection':ab,ti OR 'hospital infections':ab,ti | 13152 |
|  | #1 AND #2 | 80 |

**Web of science**

| #1 | ((TS=(Extracorporeal Membrane Oxygenation )) OR TS=(ECMO )) OR TS=(Extracorporeal Life Support) | 18585 |
| --- | --- | --- |
| #2 | ((TS=(Healthcare Associated Infection )) OR TS=(Nosocomial Infection)) OR TS=(Hospital Infections) | [122,567](https://ras.cdutcm.lib4s.com:7080/s/com/webofscience/www/G.https/wos/woscc/summary/66007d66-3694-4444-91aa-2e6dcb6414c8-8ee41c4a/relevance/1) |
|  | #1 AND #2 | 681 |

**Web of science**

| #1 | (Extracorporeal Membrane Oxygenation):ti,ab,kw OR (ECMO ):ti,ab,kw OR (Extracorporeal Life Support):ti,ab,kw | 1409 |
| --- | --- | --- |
| #2 | (Healthcare Associated Infection):ti,ab,kw OR (Nosocomial Infection):ti,ab,kw OR (Hospital Infections):ti,ab,kw | 31334 |
|  | #1 AND #2 | 265 |
